# MGUS Predicts Worse Prognosis in Patients with Coronary Artery Disease

**DOI:** 10.1007/s12265-019-09950-w

**Published:** 2020-01-03

**Authors:** Zhao Xu, Yifeng Sun, Tianhong Xu, Yidan Shi, Lifan Liang, Peng Liu, Junbo Ge

**Affiliations:** 1 Department of Hematology, Zhongshan Hospital Fudan University, 180 Fenglin Road, Shanghai, 200032 China; 2 Department of Statistics and Actuarial Science, University of Waterloo, Waterloo, Canada; 3 Department of Cardiology, Shanghai Institute of Cardiovascular Diseases, Zhongshan Hospital Fudan University, Shanghai, China

**Keywords:** MGUS, Prognosis, Coronary artery disease, Nomogram

## Abstract

We performed a retrospective cohort study to analyze all 87 CAD patients with MGUS and 178 CAD patients without MGUS admitted in Zhongshan Hospital Fudan University from 2015 to 2017. Patients were followed up via regular patient visits or telephone, and the median follow-up period was 2.9 years. The end point of follow-up was the occurrence of major adverse cardiac events (MACE). CAD patients with MGUS had a higher risk of MACE than those without MGUS (log-rank *P* = 0.0015). After adjustment for other markers in the stepwise Cox regression model, MGUS was still related to the increasing risk of MACE incident (*P* = 0.002, HR = 2.308). Then, we constructed the nomogram based on the Cox regression model, and the concordance index (C-index) was 0.667. Hence, MGUS might be added into the risk model of CAD.

**Electronic supplementary material:**

The online version of this article (10.1007/s12265-019-09950-w) contains supplementary material, which is available to authorized users.

## Introduction

Plasma cell disorders, also called monoclonal gammopathy, are verified to be premalignant and malignant disorders characterized by monoclonal proteins secreted by clonal plasma cells and immunological homogeneity on electrophoresis. Plasma cell disorders consist of many pathological conditions, including monoclonal gammopathy of undetermined significance (MGUS), solitary plasmacytoma, multiple myeloma (MM), amyloidosis, Waldenström macroglobulinemia (WM), and POEMS syndrome. Among them, MGUS is the most common disease. According to the research from Mayo Clinic in 2012 [1], 51% of patients diagnosed with monoclonal gammopathy were actually proved to be MGUS patients. MGUS was defined as monoclonal proteins in the serum < 30 g/L and clonal plasma cells in bone marrow < 10% without any myeloma-related symptoms [2]. The progression rate of MGUS to multiple myeloma is 1% per year [3, 4].

In our hospital, serum protein electrophoresis (SPEP), the monoclonal-protein screening test, is routinely incorporated into liver biochemical and function tests. Hence, all patients having liver function examinations are indeed screened for monoclonal proteins simultaneously in our institute. With routine SPEP screening, we have the unique opportunity to observe the prevalence of monoclonal gammopathy in the large-scale hospital population and to identify patients with MGUS. If the abnormal band is found via SPEP, physicians should perform the serum immunofixation electrophoresis (IFE) to confirm the existence, the concentration, and the type of monoclonal proteins [5]. To accomplish differential diagnosis, hematologists also suggest blood routine examinations, renal function tests, ionic concentrations, skeletal radiography, and bone marrow examination if necessary.

As a geriatric disease, the diagnosis of MGUS might be simultaneously established with coronary artery disease (CAD). In our daily clinical work, we found a surprising phenomenon that these patients seemed to experience worse outcomes than CAD patients without MGUS. The hypothesis was supported by the abnormal ratio of serum free light chain (FLC) in MGUS patients [6]. High FLC ratio was reported to correlate with severe cardiac involvements in patients suffering from amyloidosis [7–9] and secondary heart failure [10]. This may support the proposal in some degree. Besides, MGUS is also associated with high blood viscosity [11], which may play a role in the nosogenesis of CAD [12–14]. Therefore, the objective of our research was to clarify the relationship between MGUS and CAD and whether MGUS is an independent prognostic indicator for CAD patients.

## Methods

**Fig. 1:**
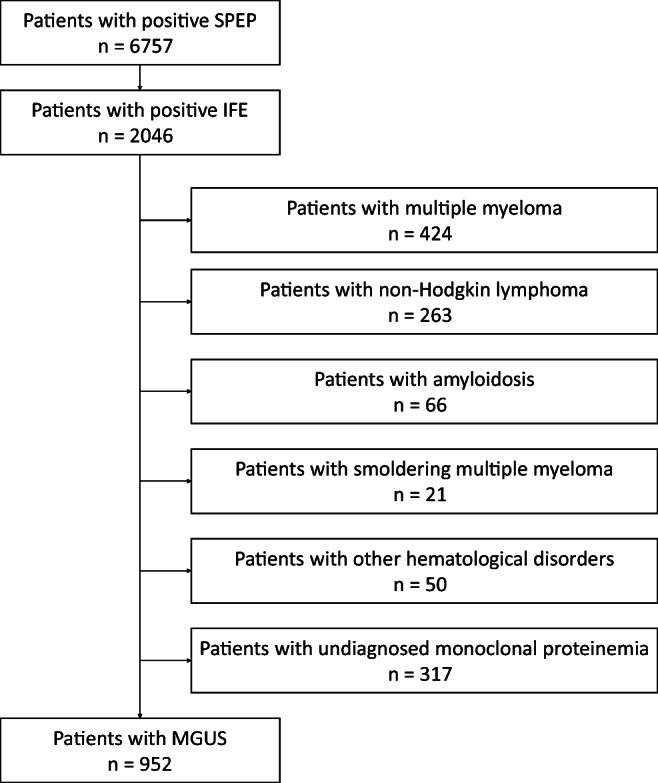
Monoclonal-protein based MGUS screening protocol. MGUS refers to monoclonal gammopathy of undetermined significance; SPEP, serum protein electrophoresis; IFE, immunofixation electrophoresis

We enrolled all inpatients and outpatients (*n* = 6757) with positive SPEP from January 1, 2015 to December 31, 2017 at Fudan University affiliated to Zhongshan Hospital. The serum IFE was performed to confirm the presence of monoclonal gammopathy in these patients. Among these patients (*n* = 2046) with positive IFE, the diagnosis of MGUS was determined according to the diagnostic criteria. Only patients with unexplained anemia (the value of hemoglobulin was less than 10 g/dL or more than 2 g/dL under the inferior limit of normal scope) or renal insufficiency (serum creatinine was more than 177 μmol/L) [15] were required to have bone examination to exclude multiple myeloma or related disorders [16]. The complete monoclonal-protein-based MGUS screening protocol in our hospital was shown in Fig. 1. Among these MGUS patients (*n* = 952), some (*n* = 87) also suffered from CAD confirmed by the coronary angiogram.

A random number function were used to select half a month (January 1, 2015, to January 15, 2015) from 3 years (2015–2017), and all CAD patients (*n* = 178) without MGUS were enrolled during this period at Zhongshan Hospital. The exclusion criteria were the absence of coronary angiogram, non-obstructive CAD patients, and coronary angiography showing less than 50% stenosis without a history of coronary revascularization.

These CAD patients were followed up via regular patient visits and telephone. The median follow-up period was 2.9 years until September 2018. The end point was the occurrence of myocardial infarction, stroke, coronary revascularization, and all-cause mortality, which was also called the major adverse cardiac events (MACE).

Clinical and biochemical information of these patients were collected, including age, gender, diagnosis, smoking status, history of CAD, stent numbers, ejection fraction, the type, and concentration of monoclonal protein; values of serum glucose, Hb1Ac, estimated glomerular filtration rate (eGFR), cardiac troponin T (cTNT), low-density lipoprotein (LDL), C-reactive protein (CRP), N-terminal pro-brain natriuretic peptide (NT-proBNP), and coagulation function containing activated partial thromboplastin time (APTT), prothrombin time (PT), and fibrinogen. Among them, numbers of stent were to evaluate the severity of CAD. Informed consent was obtained from all patients in Zhongshan Hospital. Fudan University affiliated Zhongshan Hospital Ethics Committee approved this study, and our research was conformed to the principles outlined in the Declaration of Helsinki.

The consecutive variables were analyzed by Mann-Whitney U test or t-test. Categorical outcomes were compared through chi-square test. The multiple linear regression models were performed to investigate the impact of MGUS on NT-proBNP and cTNT, which were demonstrated as the most important markers in prognosis of CAD patients [17–20]. The association between MGUS and outcomes was shown via Kaplan-Meier curves. Then, multivariate stepwise Cox regression was performed, and the model with lowest Akaike information criterion (AIC) was selected as the best to describe the outcomes of CAD patients. CRP was not included in the Cox model due to the large data missing. Next, the nomogram based on the Cox regression was constructed, and concordance index (C-index) was to evaluate its discrimination, with the value of 0.5 manifesting no discrimination and 1.0 manifesting perfect discrimination between two random patients [21]. Calibration curve was plotted to evaluate the accuracy of the nomogram by comparing the predicted and actual non-MACE probability.

All statistical tests were two-sided, and the analysis was made by SPSS 21 software (IBM Corp. Released 2012. Armonk, NY: IBM Corp.) and R software, Version 3.6.0 (R Core Team, R Foundation for Statistical Computing). *P* < 0.05 was regarded as statistical significance.

## Results

All 952 MGUS patients at Zhongshan Hospital from January 1, 2015 to December 31, 2017 were enrolled. The participants included 649 males and 303 females, and the median age was 66 (range, 19–97 years old). Among them, 19 (2.0%) of the patients had light chain MGUS, 629 (66.1%) had IgG MGUS, 92 (9.7%) had IgM MGUS, 178 (18.7%) had IgA MGUS, and 34 (3.6%) had biclonal gammopathy or triclonal gammopathy. About 769 patients with MGUS had concomitant diseases, including 384 cardiovascular diseases (267 hypertension, 87 coronary artery disease confirmed by coronary angiogram), 193 gastrointestinal and hepatobiliary diseases (44 hepatitis, 41 hepatic tumor), 145 urinary diseases (54 chronic kidney disease), 131 respiratory diseases (44 chronic obstructive pulmonary disease, 37 lung cancer), and so on.

Table 1 gave the baseline features and comparison of the clinical and biochemical characteristics between CAD patients with and without MGUS. Gender (*P* = 0.032) and age (*P* = 0.003) were demonstrated to be statistically significant, and there existed no difference of stent numbers between MGUS group and non-MGUS group, illustrating that the CAD severity of MGUS patients was similar with that of non-MGUS patients. Besides, 99 underwent coronary angiography among 952 MGUS patients, and 12 were confirmed without 50% stenosis and 87 with at least 50% stenosis. When it comes to 199 non-MGUS patients experiencing coronary angiography, 21 were confirmed without 50% stenosis and 178 with at least 50% stenosis. There existed no difference in the frequency of patients who underwent angiography but did not have significant stenosis between MGUS group and non-MGUS group (*P* = 0.165), also demonstrating that MGUS patients and non-MGUS patients had similar CAD severity.

**Table 1:** Basic characteristic of CAD patients with and without monoclonal gammopathy of undetermined significance (MGUS)

Characteristics	MGUS	Non-MGUS	*P*
Categorical Variables			
Male gender	80(92.0%)	146(82.0%)	0.032
Hypertension	63(72.4%)	112(62.9%)	0.125
Diabetes	21(24.1%)	58(32.6%)	0.158
Smoking	35(40.2%)	79(44.4%)	0.472
History of CAD	47(54.0%)	106(59.6%)	0.392
Consecutive variables			
Age	68.39	64.13	0.003
Stent Numbers	2.00	2.56	0.174
eGFR	79.02	82.08	0.426
LDL	1.91	2.08	0.089
CRP	6.12	6.85	0.555
cTNT	0.26	0.47	0.511
NT-proBNP	1474.61	415.69	0.123
PT	11.82	11.59	0.259
APTT	28.72	29.24	0.088
Fibrinogen	283.83	275.24	0.481

**Fig. 2:**
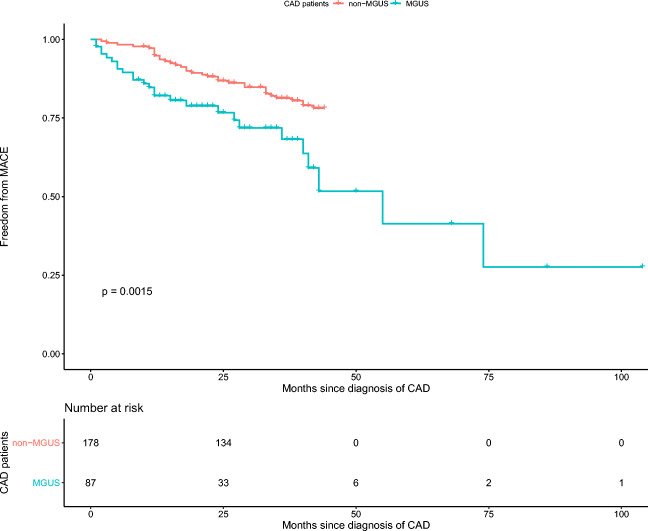
Kaplan-Meier survival curves for incidence of MACE in CAD patients with and without MGUS. MACE refers to major adverse cardiac events; CAD, coronary artery disease; MGUS, monoclonal gammopathy of undetermined significance

In our research, all 265 CAD patients (87 with MGUS and 178 without MGUS) were followed up with the median period of 2.9 years, and 60 MACE incidents were observed, including 4 cases of myocardial infarction, 42 cases of coronary revascularization, 12 cases of deaths, and 2 cases of stroke. The results indicated that the CAD patients with MGUS experienced a higher risk of MACE than those without MGUS (log-rank *P* = 0.0015) (Fig. 2). The median follow-up time of non-MGUS group was 43 months, and the interquartile range of follow-up time for non-MGUS group was from 25 months to 44 months; the median follow-up time of MGUS group was 20 months, and the interquartile range of follow-up time for non-MGUS group was from 11 months to 34 months. Based on the prognosis models of CAD mentioned in other articles [1, 2, 12], NT-proBNP and cTNT were considered as the most important factors. Our research interests lay on whether MGUS had an independent influence on the NT-proBNP and cTNT. Linear regression models were performed to investigate the impact of MGUS on NT-proBNP and cTNT, and the results were given in Table S1, indicating that MGUS was statistically significant correlated with NT-proBNP (β = 0.152, *P* = 0.022) after eliminating the influence of other high-risk factors in CAD patients.

To quantify the effects of risk factors on the incidence of MACE and to clarify the independent influence of MGUS, the multivariate stepwise Cox regression model with the minimum AIC (Table 2) was performed on the same sample as in Fig. 2. Hazard ratios (HR) and *p* values were shown, indicating that MGUS (*P* = 0.002), stent numbers (*P* = 0.045), eGFR (*P* = 0.023), and fibrinogen (*P* = 0.049) were independently related to the outcomes of CAD patients. CAD patients with MGUS had significant higher probability of experiencing MACE (*P* = 0.002, HR = 2.308, 95%CI = [1.351, 3.943]) compared with those without MGUS even after eliminating the influence of other risk factors, including gender and age.

**Table 2:** Results of unadjusted and adjusted Cox model focusing on the risk of MACE in CAD patients

	Unadjusted β coefficient	Unadjusted *P* value	Unadjusted HR (95% CI)	Adjusted β coefficient	Adjusted *P* value	Adjusted HR (95% CI)
MGUS	0.837	0.002	2.309 (1.356,3.934)	0.836	0.002	2.308 (1.351, 3.943)
Stent numbers	0.083	0.117	1.086 (0.980,1.204)	0.103	0.045	1.109 (1.002, 1.226)
eGFR	− 0.017	0.017	0.983 (0.969, 0.997)	− 0.017	0.023	0.984 (0.970, 0.998)
Fibrinogen	0.004	0.028	1.004 (1.000, 1.007)	0.003	0.049	1.003 (1.000, 1.007)

**Fig. 3:**
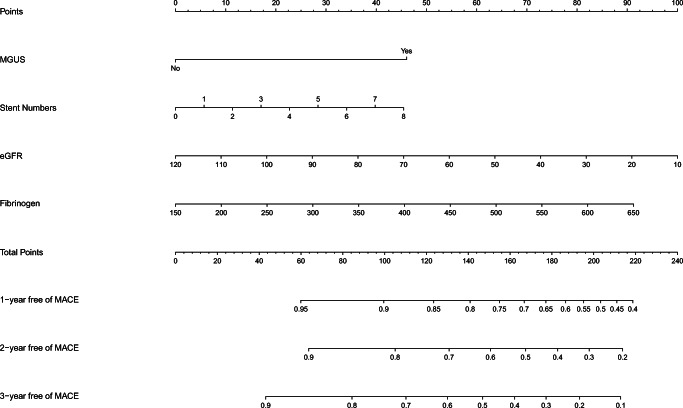
The nomogram based on the stepwise Cox regression model to predict the outcomes of CAD patients. MGUS refers to monoclonal gammopathy of undetermined significance; eGFR, estimated glomerular filtration rate; MACE, major adverse cardiac events

**Fig. 4:**
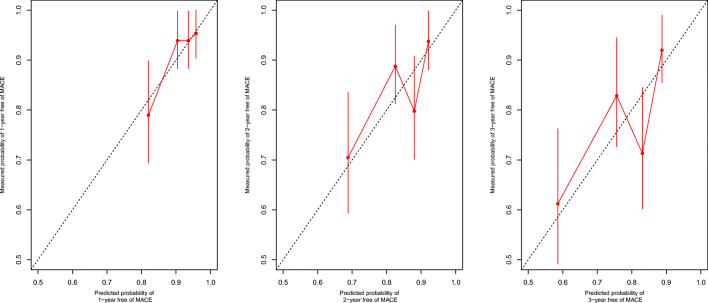
The calibration curve of MACE-free survival at 1, 2 and 3 years for CAD patients. Nomogram-predicted MACE-free probability is plotted on the x-axis; actual MACE-free probability is plotted on the y-axis

Then, the nomogram (Fig. 3) was generated to predict 1-, 2-, and 3-year non-MACE probability based on the statistically significant variables in the multivariate stepwise Cox regression model. First, we could match the score of each factor in the nomogram and added them together to get the total points. Then, drew the vertical line from the scale of total points to obtain 1-, 2-, and 3-year non-MACE probability. The C-index of the nomogram was 0.667 (95%CI, 0.592–0.742) indicating good discrimination of the nomogram. The calibration curve (Fig. 4) demonstrated that it was more accurate to predict non-MACE probability of 1-year than that of 2- and 3-year.

## Discussion

To our knowledge, this is the first research to illustrate the role of MGUS in the prognosis of CAD patients. The first important finding was that CAD patients with MGUS had worse prognosis than those without MGUS. The second significant finding was that MGUS had an independent influence on the value of NT-proBNP (*P* = 0.022), which was confirmed to be one of the most important risk factors in the coronary artery disease [22–26]. Furthermore, MGUS still remained to be an independent predictive factor of MACE incidents in the stepwise Cox regression model, and the hazard ratio was 2.308 compared to those without MGUS after eliminating the influence of other risk factors. The influence of obesity could usually be explained by the presence of hypertension or diabetes; therefore, we did not incorporate body mass index into the independent variables.

The nomogram described in results was considered as model 1 (Fig. 3), and two known most important risk factors (cTNT and NT-proBNP) were added into model 1 to construct model 2 (Fig. S1). The C-index of model 2 was 0.672 (95%CI, 0.597–0.747), and the difference of C-index between model 1 and model 2 was not statistically significant (*P* = 0.165). It was more accurate to predict non-MACE probability of 2- and 3-year using model 2, and the predictive accuracy was similar in 1-year prediction (Fig. S2). The decision curve analysis of both model 1 and model 2 (Fig. S3) indicated that model 2 showed a better net benefit after 1-year and 2-year than model 1. However, net reclassification improvement (NRI) and integrated discrimination improvement (IDI) showed no significant difference between model 1 and model 2 (Table S2). The NRIs for model 2 were negative supports that reclassification with the additional covariates in model 2 made performance worse and there was no need to include cTNT and NT-proBNP into our nomogram.

It was assumed that abnormal free light chain and high blood viscosity might be two reasons why CAD patients with MGUS experienced worse prognosis. The serum free light chain (FLC) referred to the light chains (κ and λ) which were unbound to heavy chains in the serum [27], and the normal range of FLC ratio (κ/λ) was 0.26–1.65. About one third of MGUS patients had abnormal ratio of FLC [6], and serum FLC was confirmed to have an influence on patients suffering from heart failure secondary to CAD [28]. Another study reported that dilated cardiomyopathy patients with the decrease of FLC values had an improvement in the cardiac and clinical performance [29]. In our study, the FLC ratio of 17 patients was obtained, and 3 patients had abnormal FLC ratio. The MACE risk between these 3 patients with abnormal FLC and 14 patients with normal FLC was not significantly different (*P* = 0.37). Perhaps, we will be able to figure out whether there is correlation between FLC and worse prognosis of CAD patients when we get enough data in the future.

Furthermore, there was an increase in the blood viscosity in patients with MGUS compared with healthy population [11]. This phenomenon was explained by the contribution of monoclonal proteins [30]. The high concentration of monoclonal protein, especially IgM, provided a high probability of the hyperviscosity syndrome, which happened in 10–30% of WM patients and 2–6% of MM patients [31]. Besides, the high blood viscosity was shown to correlate with an increased risk and high mortality of CAD [12–14] and played a significant role in the nosogenesis of atherosclerosis. Increased viscosity led to the decreased flow velocity and reduced wall shear stress, which regulated the atherogenic gene expression and the response to intimal injury [32]. Therefore, high viscosity in MGUS patients might be another reason of worse prognosis.

Finally, MGUS becomes popular in China with the growth of aging population and improvement of detection methods. The prevalence in individuals older than 50 year old has increased to more than 4% [33–35]. In the meantime, CAD is also very common among the elderly, and based on our research, it is valuable and necessary to screen MGUS in CAD patients, and CAD patients with MGUS might need a more frequent follow-up, but it still needs further research.

## Electronic supplementary material

Fig S1 (PNG 1319 kb).

High Resolution Image (TIFF 3750 kb).

Fig S2 (PNG 3392 kb).

High Resolution Image (TIF 348 kb).

Fig S3 (PNG 1378 kb).

High Resolution Image (TIF 302 kb).

Table S1 (DOCX 14 kb).

Table S2 (DOCX 14 kb).

Table S3 (DOCX 12 kb).

## Abbreviation

CAD: coronary artery disease
MGUS: monoclonal gammopathy of undetermined significance
MACE: major adverse cardiac events
C-index: concordance index
MM: multiple myeloma
WM: Waldenström macroglobulinemia
SPEP: serum protein electrophoresis
IFE: immunofixation electrophoresis
FLC: free light chain
eGFR: estimated glomerular filtration rate
cTNT: cardiac troponin T
LDL: low-density lipoprotein
CRP: C-reactive protein
NT-proBNP: N-terminal pro-brain natriuretic peptide
APTT: activated partial thromboplastin time
PT: prothrombin time
AIC: Akaike information criterion
HR: hazard ratios
NRI: net reclassification improvement
IDI: integrated discrimination improvement
