# Supplementary figures and images for: MGUS Predicts Worse Prognosis in Patients with Coronary Artery Disease
Source: J Cardiovasc Transl Res. 2020 Jan 3;13(5):806–12. doi: 10.1007/s12265-019-09950-w (PMC7541390; doi:10.1007/s12265-019-09950-w)

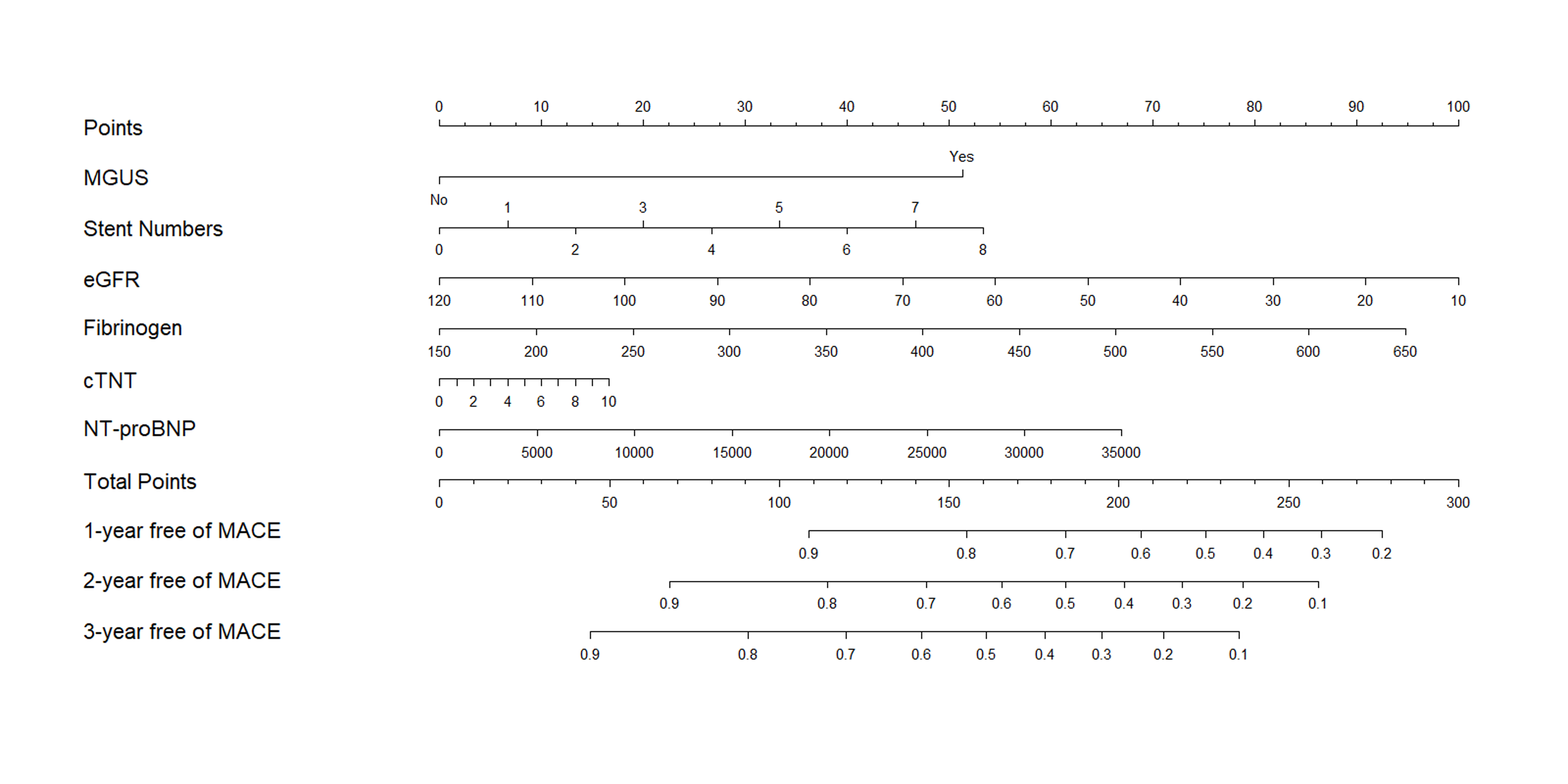

Supplement: Supplementary file 1 — (PNG 1319 kb). [file 12265_2019_9950_Fig5_ESM.png]

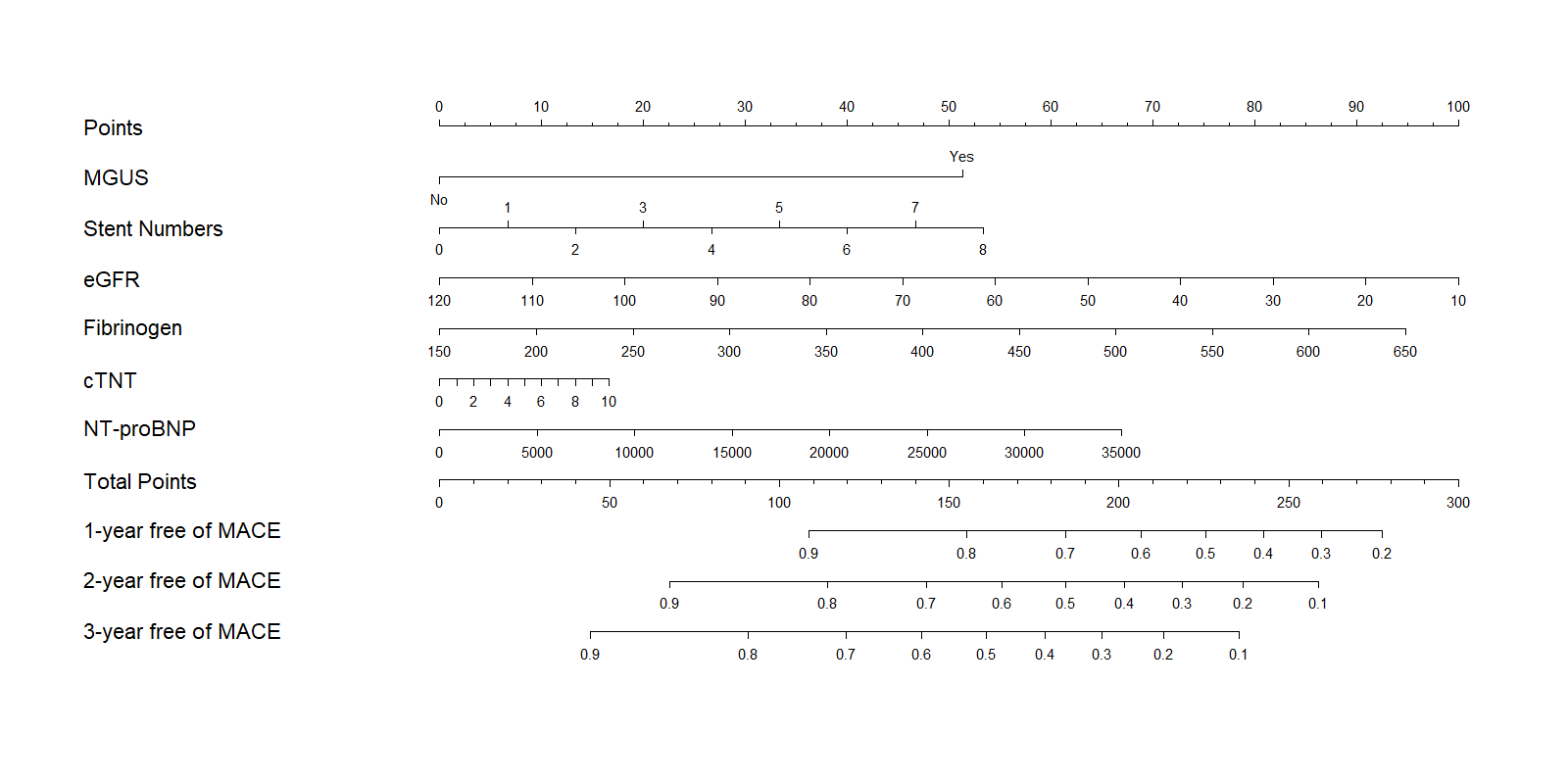

Supplement: Supplementary file 2 — High Resolution Image (TIFF 3750 kb). [file 12265_2019_9950_MOESM1_ESM.tiff]

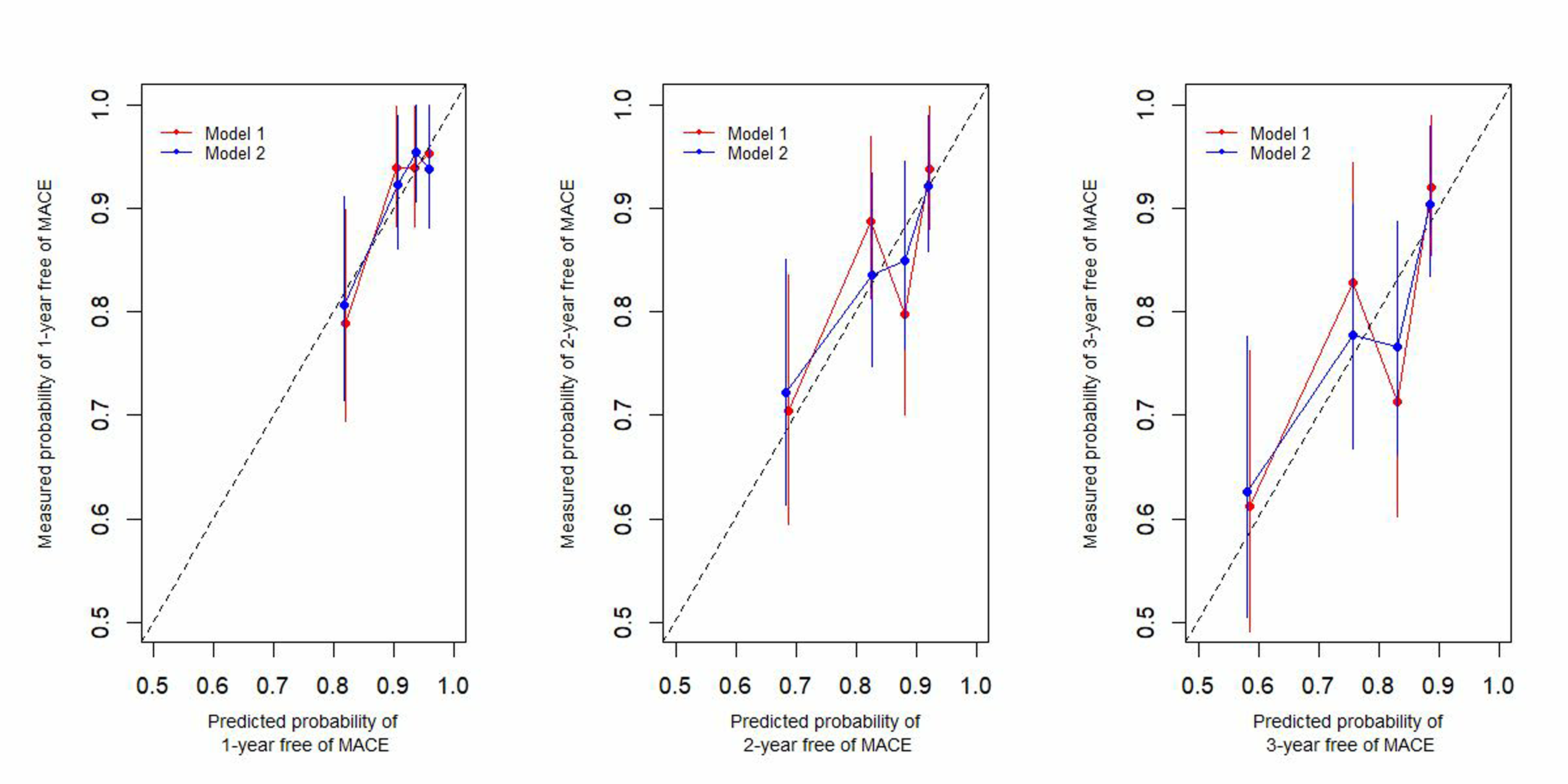

Supplement: Supplementary file 3 — (PNG 3392 kb). [file 12265_2019_9950_Fig6_ESM.png]

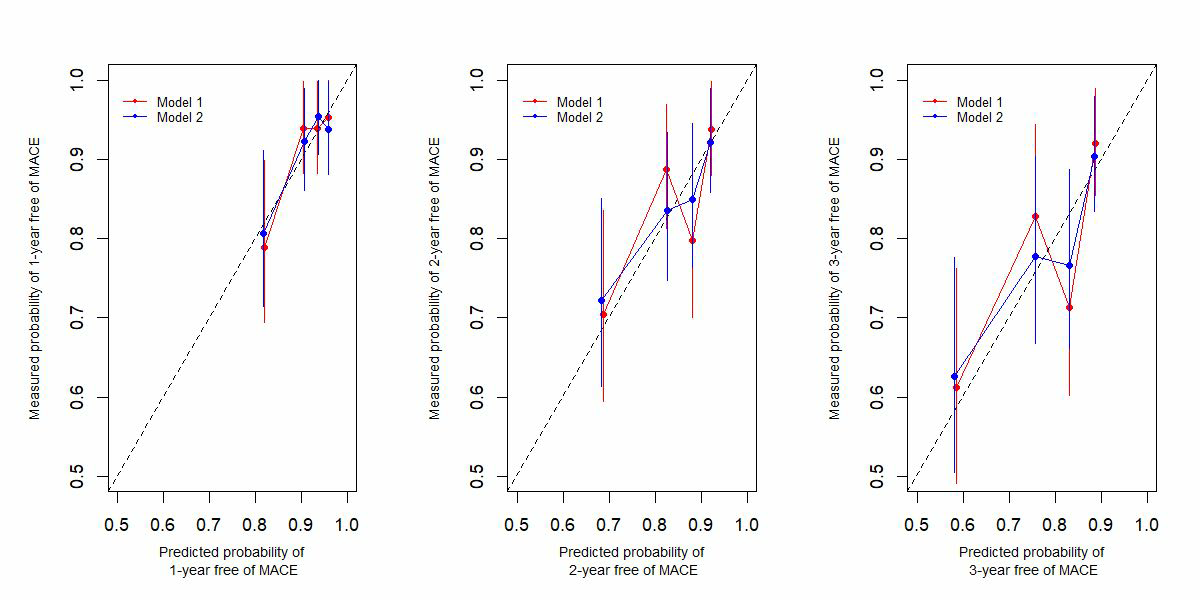

Supplement: Supplementary file 4 — High Resolution Image (TIF 348 kb). [file 12265_2019_9950_MOESM2_ESM.tif]

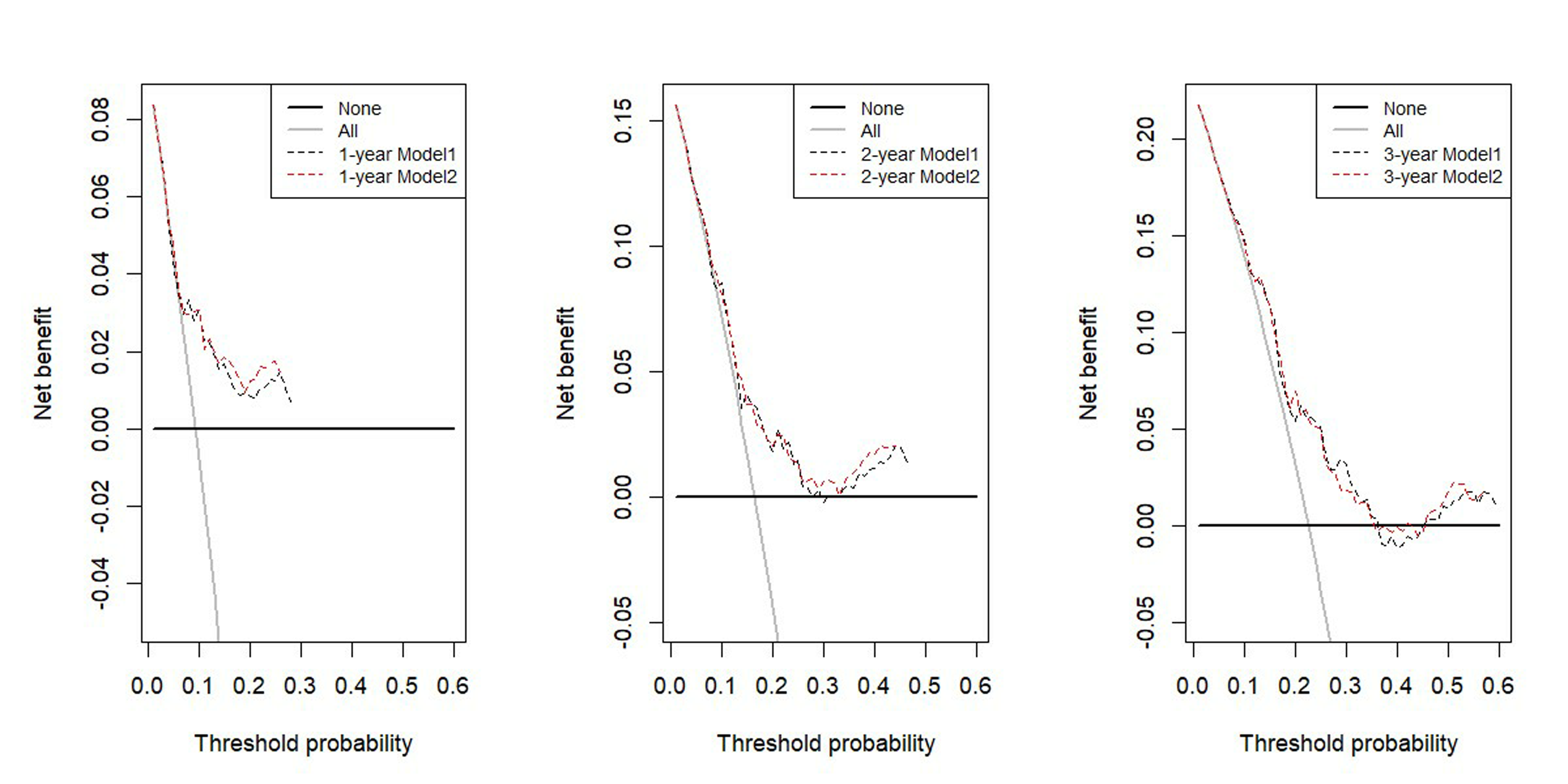

Supplement: Supplementary file 5 — (PNG 1378 kb). [file 12265_2019_9950_Fig7_ESM.png]

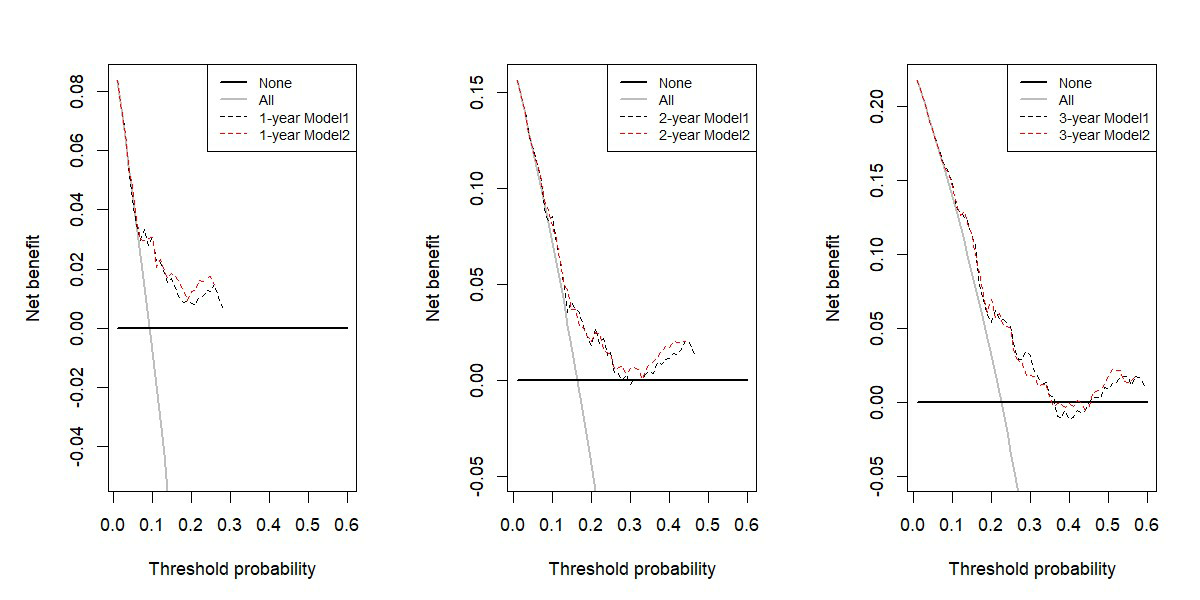

Supplement: Supplementary file 6 — High Resolution Image (TIF 302 kb). [file 12265_2019_9950_MOESM3_ESM.tif]
