# Supplementary material for: MGUS Predicts Worse Prognosis in Patients with Coronary Artery Disease
Source: J Cardiovasc Transl Res. 2020 Jan 3;13(5):806–12. doi: 10.1007/s12265-019-09950-w (PMC7541390; doi:10.1007/s12265-019-09950-w)
Supplement: Supplementary file 7 — (DOCX 14 kb). [file 12265_2019_9950_MOESM4_ESM.docx]

| **Variables** | **cTNT** | | **NT-proBNP** | |
| --- | --- | --- | --- | --- |
|  | **β** | ***P*** | **β** | ***P*** |
| Male gender | -0.035 | 0.609 | -0.012 | 0.862 |
| Age | -0.098 | 0.126 | 0.097 | 0.138 |
| MGUS | -0.008 | 0.898 | 0.152 | 0.022 |
| Smoking | 0.138 | 0.039 | 0.047 | 0.493 |
| Hypertension | 0.014 | 0.825 | 0.008 | 0.906 |
| Diabetes | -0.046 | 0.466 | 0.025 | 0.695 |
| LDL | 0.188 | 0.004 | -0.043 | 0.507 |

**Table S1 Multiple linear regression of cTNT and NT-proBNP in CAD patients.**
