# Supplementary material for: MGUS Predicts Worse Prognosis in Patients with Coronary Artery Disease
Source: J Cardiovasc Transl Res. 2020 Jan 3;13(5):806–12. doi: 10.1007/s12265-019-09950-w (PMC7541390; doi:10.1007/s12265-019-09950-w)
Supplement: Supplementary file 8 — (DOCX 14 kb). [file 12265_2019_9950_MOESM5_ESM.docx]

|  | **Estimates** | ***P*** | **95%CI** |
| --- | --- | --- | --- |
| 1-year IDI | 0.000 | 0.850 | (-0.012, 0.025) |
| 1-year NRI | -0.132 | 0.817 | (-0.240, 0.250) |
| 2-year IDI | 0.000 | 0.405 | (-0.061, 0.002) |
| 2-year NRI | -0.070 | 0.598 | (-0.206, 0.164) |
| 3-year IDI | 0.000 | 0.432 | (-0.027, 0.002) |
| 3-year NRI | -0.104 | 0.824 | (-0.225, 0.186) |

**Table S2 net reclassification improvement (NRI) and integrated discrimination improvement (IDI) of model 1 and model 2.** The NRIs for Model 2 were negative supports that reclassification with the additional covariates in Model 2 made performance worse.
