# Supplementary material for: MGUS Predicts Worse Prognosis in Patients with Coronary Artery Disease
Source: J Cardiovasc Transl Res. 2020 Jan 3;13(5):806–12. doi: 10.1007/s12265-019-09950-w (PMC7541390; doi:10.1007/s12265-019-09950-w)
Supplement: Supplementary file 9 — (DOCX 12 kb). [file 12265_2019_9950_MOESM6_ESM.docx]

|  | **Beta coefficient** | ***P*** | **Odds Ratio** |
| --- | --- | --- | --- |
| **MGUS** | 0.585 | 0.062 | 1.796 |
| **Stent Numbers** | 0.133 | 0.042 | 1.142 |
| **eGFR** | -0.021 | 0.018 | 0.979 |
| **Fibrinogen** | 0.004 | 0.100 | 1.004 |

**Table S3** the logistic regression model based on the same variables in the Cox regression model.
